# The role of plasma membrane STIM1 and Ca^2 +^entry in platelet aggregation. STIM1 binds to novel proteins in human platelets

**DOI:** 10.1016/j.cellsig.2013.11.025

**Published:** 2014-03

**Authors:** A. Ambily, W.J. Kaiser, C. Pierro, E.V. Chamberlain, Z. Li, C.I. Jones, N. Kassouf, J.M. Gibbins, K.S. Authi

**Affiliations:** a Cardiovascular Division, BHF Centre for Research Excellence, King's College London, Franklin Wilkins Building, Stamford Street, London SE1 9NH, United Kingdom; b Institute of Cardiovascular and Metabolic Research and School of Biological Sciences, University of Reading, Reading, United Kingdom

**Keywords:** STIM1, stromal interaction molecule 1, SOCE, store operated Ca^2 +^ entry, DAG, 1,2-diacyl-sn-glycerol, PM, plasma membrane, TG, thapsigargin, OAG, 1-Oleoyl-2-acetyl-*sn*-glycerol, TRPC, transient receptor potential canonical, Aggregation, Ca^2 +^ entry, Collagen, STIM1, Thrombospondin-1

## Abstract

Ca^2 +^ elevation is essential to platelet activation. STIM1 senses Ca^2 +^ in the endoplasmic reticulum and activates Orai channels allowing store-operated Ca^2 +^ entry (SOCE). STIM1 has also been reported to be present in the plasma membrane (PM) with its N-terminal region exposed to the outside medium but its role is not fully understood. We have examined the effects of the antibody GOK/STIM1, which recognises the N-terminal region of STIM1, on SOCE, agonist-stimulated Ca^2 +^ entry, surface exposure, in vitro thrombus formation and aggregation in human platelets. We also determined novel binding partners of STIM1 using proteomics. The dialysed GOK/STIM1 antibody failed to reduced thapsigargin- and agonist-mediated Ca^2 +^ entry in Fura2-labelled cells. Using flow cytometry we detect a portion of STIM1 to be surface-exposed. The dialysed GOK/STIM1 antibody reduced thrombus formation by whole blood on collagen-coated capillaries under flow and platelet aggregation induced by collagen. In immunoprecipitation experiments followed by proteomic analysis, STIM1 was found to extract a number of proteins including myosin, DOCK10, thrombospondin-1 and actin. These studies suggest that PM STIM1 may facilitate platelet activation by collagen through novel interactions at the plasma membrane while the essential Ca^2 +^-sensing role of STIM1 is served by the protein in the ER.

## Highlights

• STIM1 promotes collagen induced platelet aggregation and thrombus formation.
• In human platelets SOCE activates but is not essential for platelet aggregation.
• Plasma membrane STIM1 may facilitate platelet activation independent of SOCE.

## 1 Introduction

Platelet activation is essential in haemostasis and plays a key role in thrombosis. Important for platelet activation is Ca^2 +^ elevation in the cytosol, which occurs as a result of release from intracellular stores and entry from the outside medium [1,41]. Whilst Ca^2 +^ release from intracellular stores by agonist-induced formation of inositol 1,4,5-trisphosphate is established, Ca^2 +^ entry mechanisms are less understood. In platelets, three pathways for Ca^2 +^ entry have been identified. Platelets express the P2X1 receptor that is a ligand-gated ion channel for ATP [27]. Ca^2 +^ entry can occur after store depletion (referred to as store-operated Ca^2 +^ entry [SOCE]) [1,41] and, second messengers (such as 1,2-diacyl-sn-glycerol [DAG]) may gate plasma membrane (PM) cation channels such as the transient receptor potential canonical 6 [TRPC6] [17,18].

Recent studies have provided major insights into the mechanisms and molecular components of SOCE. Stromal interaction molecule 1 (STIM1), a ~ 80 kDa transmembrane protein abundant in the endoplasmic reticulum (ER), is the Ca^2 +^ sensor of the ER. Upon store depletion, Ca^2 +^ comes off its EF hand domain and the protein oligomerises and translocates to *punctae* to activate PM cation entry channels [25,34]. Orai1, which belongs to a family of Orai proteins (Orai1, Orai2 and Orai3), is established as the Ca^2 +^ release activated Ca^2 +^ (CRAC) channel of haematopoietic cells [10,42]. Recently, both STIM1 and Orai1 have been shown to be essential for SOCE in platelets as the absence of either in mouse platelets leads to lack of SOCE, greatly reduced agonist-stimulated Ca^2 +^ entry and a marked protection against thrombus formation in a number of in vivo models of thrombosis but whilst aggregation responses are largely maintained [7,12,40]. The STIM1–Orai1 axis may thus represent a major target for anti-thrombotic therapy [2].

STIM1 was originally identified as a PM protein involved in pre-B cell interaction and as a regulator of cell growth [29,31,43]. An antibody recognising the N-terminal domain of STIM1 (GOK/STIM1) has been reported to inhibit SOCE in intact HEK-293 cells [35] and in intact platelets [26] suggesting that some STIM1 is present in the PM with the EF-hand domain exposed on the outer surface. However in other studies STIM1 has been proposed not to be expressed in the PM, but to translocate to regions of juxtaposition to the PM upon activation [25]. To examine these issues we have studied possible functions of surface-exposed STIM1 in human platelets. We report that, the purified STIM1 antibody failed to inhibit Ca^2 +^ elevation by store depletion and by agonists in human platelets. However the antibody reduced thrombus formation by human blood on collagen-coated capillaries under flow and platelet aggregation to collagen. Proteomic analysis of immunoprecipitated STIM1 revealed the protein to bind to myosin, actin, DOCK10 and thrombospondin-1. Our studies suggest that PM STIM1 may take part in novel interactions at the plasma membrane supporting platelet aggregation but that SOCE is not essential for aggregation in human platelets.

## 2 Materials and methods

### 2.1 Reagents

Unless stated otherwise, reagents were purchased from Sigma Aldrich (Dorset, UK). The GOK/STIM1 antibody and control mouse IgG2a were from BD Biosciences (Oxford, UK). PL/IM 430 (used as a control antibody, recognises SERCA3) and PM6/40 (recognising GP1B) were purified from hybridoma cell cultures as previously described [6]. Polyclonal STIM1 antibody recognising a C-terminal epitope was from ProSci (Poway, USA). IID8 antibody to SERCA 2 was purchased from Abcam (Cambridge, UK). Myosin-9 and Thrombospondin-1 antibodies were from Santa Cruz (USA). BTP-2 (N-(4-[3,5-bis(trifluoromethyl)-1H-1yl]phenyl)-4-methyl-1,2,3-thiodiazole-5-carboxamide) was from Calbiochem (Nottingham, UK). LOE-908 (3,4-dihydro-6,7-dimethoxy-a-phenyl-N,N-bis[2-(2,3,4-trimethoxyphenyl)ethyl]-1-isoquinolineacetamide hydrochloride) was from Tocris (Bristol, UK).

Dialysis membranes (Membra-Cel MD10-14 × 100 CLR) were boiled for 10 min in 2% sodium bicarbonate containing 0.05% EDTA followed by boiling in double-distilled water for 5 min. Antibodies were dialysed in 2 changes of ice cold PBS overnight at 4 °C.

### 2.2 Human platelet preparation, Ca^2 +^ measurements, aggregation and flow cytometry studies

Blood was taken from healthy volunteers as stipulated by local ethical guidelines into one tenth volume 3.2% trisodium citrate. Platelet rich plasma (PRP) was prepared by centrifugation of the blood at 200 ×*g* for 20 min. Fura2-AM labelling was carried out in PRP as previously described [17] and the platelets were re-suspended at a cell count of 8 × 10^8^ cells/ml in HEPES-Tyrode buffer consisting of 10 mM Hepes, 140 mM NaCl, 5 mM KCl, 1 mM MgCl_2_, 5 mM glucose, 0.42 mM NaH_2_PO_4_·H_2_O, 12 mM NaHCO_3_, 0.2 mM EGTA, 10 μM indomethacin and 1 U/ml apyrase. Cells were incubated for 30 min at 37 °C in the presence of 5 μg/ml of either GOK/STIM1 antibody, or control IgG (PL/IM430 in PBS) [6] or vehicle control (equal volume of PBS), followed by dilution to 2 × 10^8^ cells/ml in HEPES-Tyrode buffer without EGTA and apyrase. Fura2 emission upon excitation of the cells at 340 and 380 nm was recorded using a Cairn Optoscan spectrofluorimeter. Traces shown are representative of experiments on at least three separate platelet preparations.

For aggregation studies, platelet suspensions were incubated as above with 10 μg/ml GOK/STIM1 or control IgG2a dialysed antibodies. Platelet suspensions diluted to 2 × 10^8^ cells/ml were stimulated in a Payton dual channel aggregometer (or Biodata Pap4) in the presence of 1 mM Ca^2 +^ for at least 4 min. For experiments involving BTP2 and LOE908, platelets were resuspended at 2 × 10^8^ cells/ml and reagents added as indicated in the figures.

For analysis of PM STIM1, platelets were resuspended at 1 × 10^8^ cells/ml and incubated at 37 °C with and without thapsigargin (TG, 10 μM) for 3 min. Reactions were stopped with ice cold EDTA (10 mM final). The cells were then incubated with primary antibody at 5 μg/ml for 1 h on a rocker followed by fluorescent secondary antibody (goat anti-mouse Alexa 488, 1/50 dilution of stock containing 0.5 mg/ml) for 1 h in the dark. Cells were fixed with 1% *p*-formaldehyde and kept in the dark until analysed by flow cytometry using a Beckman Coulter Cytomics FC500 MPL flow cytometer. 10,000 events were analysed for surface labelling.

### 2.3 Western blotting

Samples of platelet lysates (after lysis of 1 × 10^9^ cells/ml) or immunoprecipitations in Laemmli sample buffer, were subjected to electrophoresis using 7% gels and separated proteins were transferred onto PVDF membrane by semi-dry blotting as previously described [17]. The membranes were blocked overnight, washed 4 times, incubated with primary antibody (1/250 dilution for GOK/STIM1 or as indicated in the fig. legends) for at least 1 h, washed, incubated with secondary antibody (e.g. goat anti-mouse usually 1/10,000) conjugated to HRP, washed again and proteins detected using ECL reagents as described [17]. Samples of hTRPC6, hTRPC3, hTRPC1, mTRPC4, mTRPC5, and mTRPC7 over-expressed in QBI(HEK)-293 cells and TRPC antibody preparation and purification were prepared as described previously [17]. Antibodies in rabbits to TRPC3 (A1978) were generated using the sequence (C)RRRLQKDIEMGMGN and to TRPC7 (1567) using the sequence (C)LNKDHLRVNKGKDI. A TRPC6 monoclonal (SL-TC6) was obtained from Novartis, Horsham U.K. Blots shown are typical of at least three on separate preparations.

### 2.4 Thrombus formation in whole blood under arterial flow

Formation of thrombi on collagen-coated capillaries was carried out using a modification of the method described by Tucker et al. [36]. Whole blood was incubated with purified antibodies at 10 μg/ml at 37 °C for 30 min followed by labelling with the fluorescent lipophilic dye 3,3′-dihexylocarbocyanine iodide (DiOC_6_; 0.5 ng/ml final) in 50% ethanol (0.05% v/v final) in the dark at 30 °C for 20 min. Glass capillaries (Camlab, Cambridge, UK) of dimensions 0.1 mm × 1 mm × 50 mm were coated with 100 μl of 100 μg/ml collagen in HEPES-Tyrode overnight at 4 °C. Prior to experiments, the capillaries were blocked with 100 μl of 1% (w/v) protease free BSA for 1 h followed by flushing with HEPES-Tyrode buffer. The antibody-treated blood was then perfused over the collagen-coated capillaries at a shear rate of 1000 s^− 1^ for 4 min at room temperature. The formed thrombi were then washed with Hepes-Tyrode buffer at the same shear rate for 8 min to remove unattached cells and the capillaries were analysed using a Leica DMIRE2 inverted confocal microscope (N PLANL 20× /0.4 objective; with 0 to 2 mm correction). Five random views were selected and Z-stack images taken from the matrix surface progressing through the thrombi every 2 μm and analysed using TCS SP2 software to calculate thrombus volume.

### 2.5 Proteomic analysis of immunoprecipitated extracts of STIM1 by liquid chromatography–mass spectrometry/mass spectrometry (LC–MS/MS)

Platelets were resuspended at 1 × 10^9^ cells/ml and incubated with TG for 1 min followed by lysis in equal volume of RIPA lysis buffer and immunoprecipitation as described in [39]. Extracted samples were run on 5–15% gradient SDS-PAGE gels followed by Colloidal Coomassie staining (see Results). Segments containing visible bands and clear areas were exposed to in-gel reduction, alkylation and digestion with trypsin using standard protocols. Peptides were extracted from the gel pieces by a series of acetonitrile and aqueous washes. The extract was lyophilised, resuspended in 23 μl of 50 mM ammonium bicarbonate and analysed by LC–MS/MS using an Ultimate LC system (Dionex, UK). Peptides were resolved using 75 μm C18 PepMap column. A 60 minute gradient of acetonitrile in 0.05% formic acid was delivered to elute the peptides at a flow rate of 200 nl/min. Peptides were ionised by electrospray ionisation using a Z-spray source fitted to a QTof-micro (Waters Corp.). The MS/MS analyses were conducted using collision energy profiles based on the mass-to-charge ratio (*m*/*z*) and the charge state of the peptide.

The mass spectral data was processed using ProteinLynx Global Server v2.2.5, and the Swiss Prot database using Mascot software v2.2 (http://www.matrixscience.com). Sequence information was obtained for all the peptides included in the results.

### 2.6 Statistical analysis

*P*-values were calculated using GraphPad Prism software.

## 3 Results

### 3.1 Purified GOK/STIM1 antibody does not inhibit Ca^2 +^ entry into platelets

**Fig. 1:**
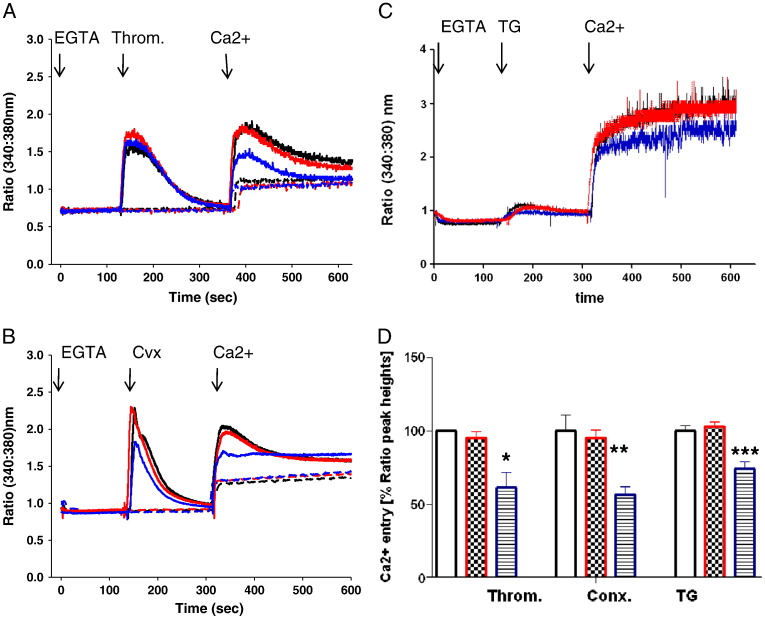

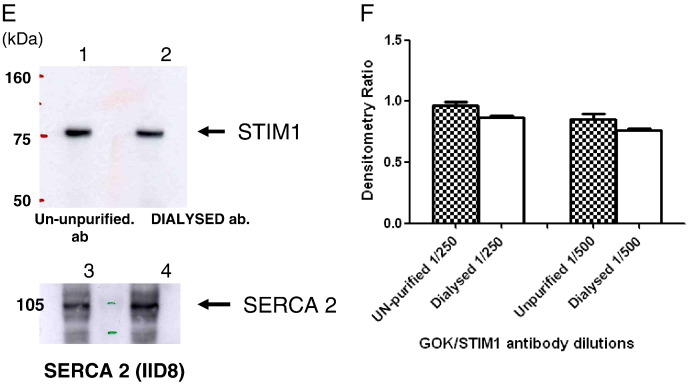
Purified GOK/STIM1 antibody does not inhibit Ca^2 +^ entry in human platelets. Fura2 labelled platelets were incubated with purified GOK/STIM1 or control PBS at 5 μg/ml for 30 min followed by analysis of Ca^2 +^ elevation using 340/380 nm ratio fluorescence. A. Platelets stimulated with thrombin (at 1.25 U/ml) in the presence of added 100 μM EGTA followed by 1 mM Ca^2 +^ addition to measure Ca^2 +^ entry. Black lines indicate incubation of platelets with control PBS, red lines with dialysed GOK/STIM1 antibody and blue lines in the presence of 0.006% sodium azide. Dotted lines in A and B indicate recordings in the absence of agonist addition but including EGTA and Ca^2 +^. Typically peak ratio increase in Ca^2 +^ entry for thrombin in the presence of PBS = 0.749 ± 0.004 [n = 3], in the presence of dialysed GOK/STIM1 = 0.714 ± 0.032 [n = 3; *P* = 0.382], in the presence of sodium azide = 0.461 ± 0.076 [n = 3, *P* = 0.038; ٭]. B. Responses in platelets stimulated by 1 μg/ml convulxin (CvX). With Ca^2 +^ release differences between the peak height were not significant (*P* = 0.09). Increase of ratio value for Ca^2 +^ entry in the presence of PBS = 0.57 ± 0.062 [n = 3], in the presence of dialysed GOK/STIM1 = 0.543 ± 0.041 [n = 3, *P* = 0.06; not significant], in the presence of sodium azide = 0.323 ± 0.031 [n = 3, *P* = 0.011; ٭٭]. C. Platelets stimulated by 3 μM TG. D. Ca^2 +^ entry expressed as % ratio peak heights after Ca^2 +^ addition for each agonist. Unfilled black bars reflect PBS control, red bars in the presence of GOK/STIM1 antibody and blue bars in the presence of sodium azide. *P = 0.03; **P = 0.01; ***P = 0.001 all compared to controls. Values are from means ± SEM (n = 3). E. Western blotting analysis of STIM1 in platelet lysates using un-purified GOK/STIM1 antibody (lane 1; 1/250 dilution) and dialysed GOK/STIM1 antibody (lane 2; 1/250 dilution). All lanes had 50 μg protein; lanes 3 and 4 were probed with SERCA 2 antibody IID8 (1/2000 dilution). F. Analysis of STIM1 recognition in platelet lysates by 2 dilutions of un-purified and dialysed GOK/STIM1 antibodies by western blotting. Detected bands were scanned by densitometry and expressed as a ratio of the recognition of SERCA 2 by IID8. *P* values were not significant.

We have recently reported that sodium azide that is present in almost all commercial antibody preparations will inhibit Ca^2 +^ entry into human platelets [39]. To examine the effects of the GOK/STIM1 antibody we dialysed the GOK/STIM1 antibody against two changes of PBS overnight and tested the antibody at a dose of 5 μg/ml (equivalent to 1/14 dilution). This was compared with a control purified antibody preparation (PL/IM430), PBS and sodium azide at a concentration of 0.006% (calculated final concentration of azide that would be present if the neat antibody was used in the experiments). Experiments were carried using the Ca^2 +^ add back protocol where platelets were incubated in 100 μM EGTA, then agonist 3 min later to examine Ca^2 +^ release from stores followed by the addition of 1 mM Ca^2 +^ 3 min later to examine Ca^2 +^ entry. The PL/IM430 control antibody (at 5 μg/ml) had no effect on basal Ca^2 +^ levels, Ca^2 +^ release from stores or Ca^2 +^ entry by agonists compared with platelets incubated with PBS (thus only traces with PBS are shown). In Fig. 1 (A,B,C) control traces in black represent agonist induced responses in the presence of PBS or PL/IM430. Incubation of platelets with 5 μg/ml GOK/STIM1 antibody showed no effect on thrombin- (1.25 U/ml) or convulxin- (1 μg/ml) or thapsigargin (TG, 3 μM) induced Ca^2 +^ release or Ca^2 +^ entry. When lower doses of thrombin were used at 0.3 and 0.6 U/ml there was also no significant effect in the presence of the GOK/STIM1 antibody (results not shown). Sodium azide however, was effective at inhibiting thrombin- and convulxin-induced Ca^2 +^ entry (by 38% [*P* = 0.038] and 43% [*P* = 0.011]) respectively (blue traces in Fig. 1 and bar charts Fig. 1D). A negative trend was observed against convulxin-stimulated Ca^2 +^ release with sodium azide but was not significant. The dialysed GOK/STIM1 was also tested at 10 and 15 μg/ml final concentration against TG induced responses but again failed to reduce Ca^2 +^ entry significantly (results not shown). However, TG-stimulated Ca^2 +^ entry was inhibited by sodium azide by 26% (*P* = 0.001) (Fig. 1).

We tested if dialysis of the GOK/STIM1 antibody may have altered the recognition of STIM1. Fig. 1E shows detection of STIM1 in Western blots using 2 dilutions of dialysed or un-dialysed antibody expressed as a ratio of the detection of SERCA 2 (used as control). No significant difference in detection of STIM1 was found (Fig. 1F). As the effects on Ca^2 +^ entry were carried out at 1/14 dilution, the lack of inhibition by the purified antibody on Ca^2 +^ entry was not due to loss of STIM1 recognition.

### 3.2 Surface expression of STIM1 by flow cytometry

**Fig. 2:**
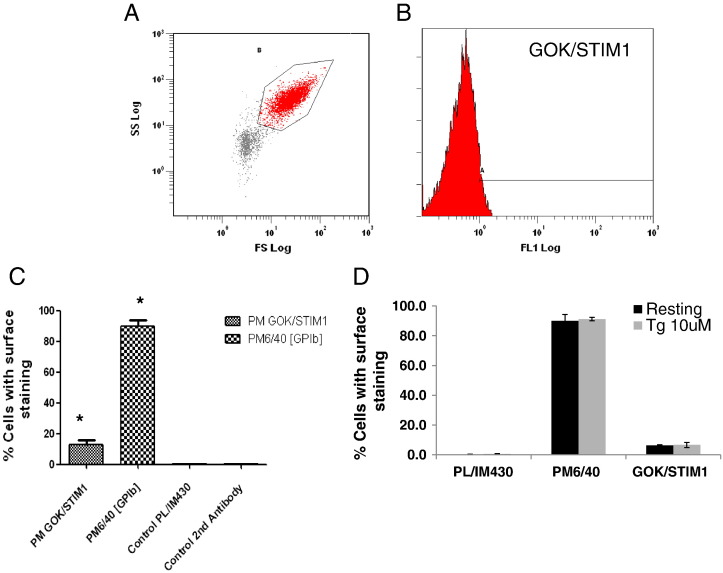
Surface expression of STIM1 using flow cytometry. Platelets were incubated with primary antibodies followed by goat anti-mouse antibody conjugated to Alexa Fluor 488 (2nd antibody). After fixation labelled cells were analysed by flow cytometry counting 10,000 events. A. Scatter plot showing gating of platelets. Particles outside of the gated area were detected in the absence of cells. B. Detection of cells labelled with GOK/STIM1 antibody (% cells stained 12.7% ± 3.2%; *P* = 0.0026) vs control second antibody (% cells stained 0.4% ± 0.15, *P* = 0.025) vs PL/IM430 (% cells stained 0.5% ± 0.06). C. Bar chart showing % cells with surface staining by respective antibodies. *P = 0.002 compared to secondary antibody control. D. Bar chart showing effects of TG treatment (10 μM) on surface expression of STIM1 (GOK/STIM1), GP1B (PM6/40) and SERCA 3 (PL/IM430). No significance was noted between TG treatments for each set (n = 3).

We next sought to determine the surface exposure of platelet STIM1 by flow cytometry of bound antibodies. Fig. 2 (A + B) shows typical gating of 10,000 platelets separated by forward and side scatter and the detection of platelets labelled with the dialysed GOK/STIM1 antibody. Bar charts show % of platelets labelled with the corresponding antibodies. Platelets incubated with PM6/40 (an antibody to GPIB) show labelling of 90% of the platelet population (Fig. 2C). With the control PL/IM430 and secondary antibodies less than 1% of cells were labelled, whereas with the GOK/STIM1 antibody the degree of labelling was approximately 13% (*P* = 0.0026 vs secondary antibody control; *P* = 0.025 vs PL/IM 430). Between donors there was a larger variation in range (from 5% to 30%) of STIM1 surface expression than for PL/IM 430 (variation 0.5–0.8%) or for PM6/40 (variation 85–99%). Following stimulation with 10 μM TG, no significant difference was observed in the labelling of platelets with the GOK/STIM1 antibody, suggesting no further insertion of STIM1 into the plasma membrane upon store depletion. Mean fluorescence intensity values of the platelets were also not different between resting or TG-treated platelets labelled with the GOK/STIM1 antibody (results not shown).

### 3.3 Effects of the GOK/STIM1 antibody on thrombus formation on collagen under flow

**Fig. 3:**
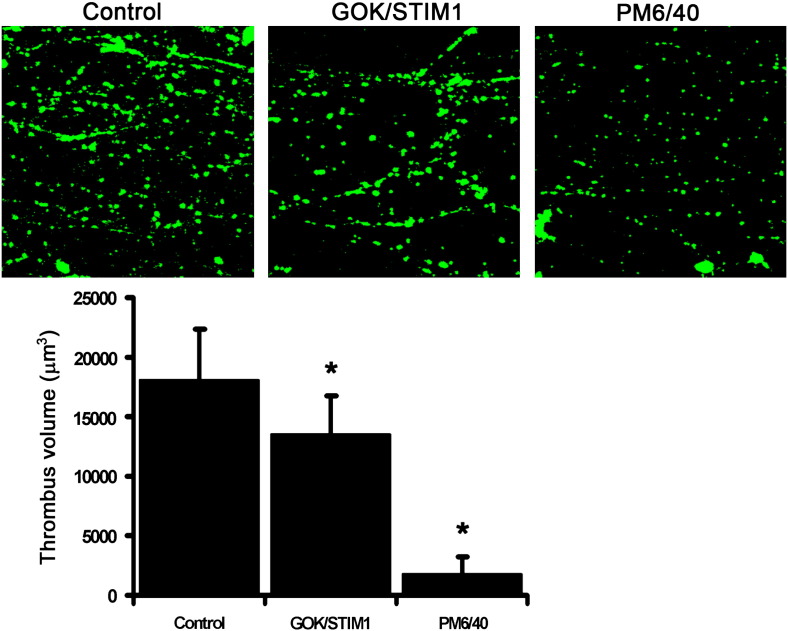
GOK/STIM1 reduces thrombus formation on collagen coated capillary under flow. Upper panel, representative images of thrombi formed on collagen coated capillaries using blood incubated with control purified antibody (PL/IM430; upper left), dialysed GOK/STIM1 antibody (centre) and dialysed GP1B antibody (PM6/40) (upper right). Bar chart, analysis of mean thrombus volume after 4 min perfusion at a shear rate of 1000 s^− 1^ (GOK/STIM1 vs control *P* = 0.048; n = 5; PM6/40 vs control *P* = 0.028; n = 3).

**Fig. 4:**
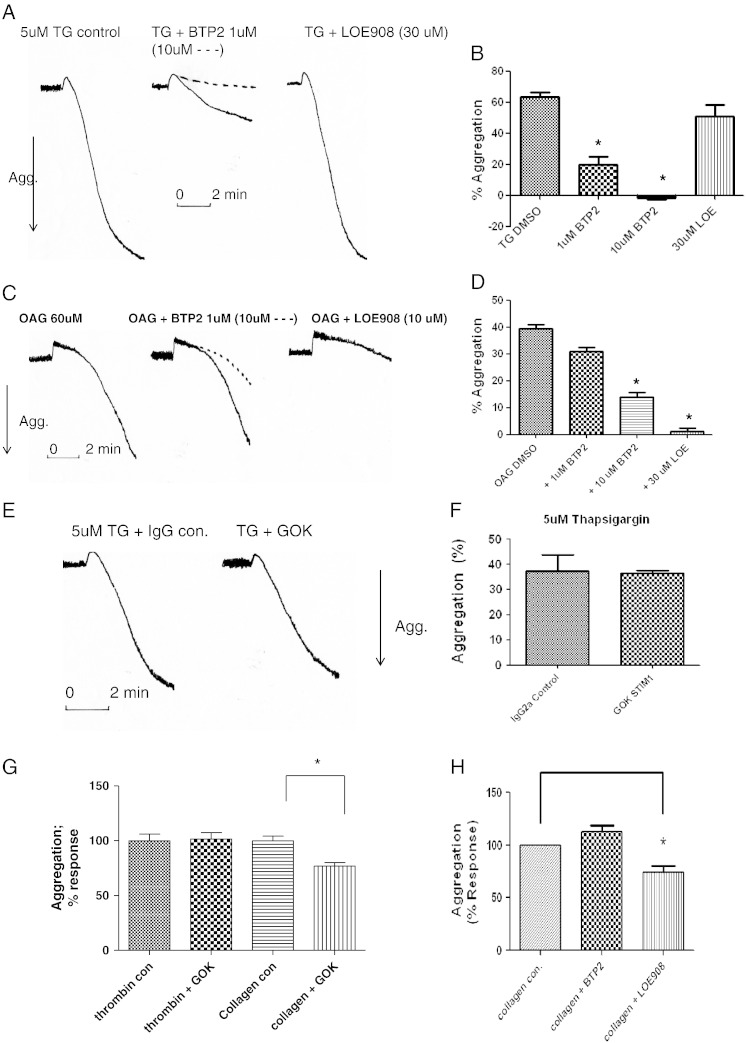
Effects of BTP2, LOE908 and the dialysed GOK/STIM1 antibody on platelet aggregation. For BTP2 and LOE908 studies the inhibitor (or vehicle) was added 5 min prior to addition of agonist (TG, OAG etc.) with 1 mM Ca^2 +^ added 3 min prior to agonist. A + B, BTP2 or LOE908 at indicated doses on TG induced platelet aggregation. C + D, BTP2 or LOE908 on OAG induced platelet aggregation. E + F, Effects of dialysed antibodies at 10 μg/ml for 30 min (GOK/STIM1 [GOK] or IgG control) on TG induced platelet aggregation. G, Effects of GOK/STIM1 on collagen (10 μg/ml) and thrombin (0.5 U/ml) induced platelet aggregation. H, Effects of BTP2 or LOE908 on collagen (15 μg/ml) induced platelet aggregation. Where shown traces are typical of six determinations. Arrow down reflects increased light transmission (aggregation) set with buffer at 100% and platelet suspension at 0%. * reflects *P* < 0.05.

We then sought to determine if PM STIM1 played any role in a model of platelet-mediated thrombus formation on collagen under flow that has been previously characterised [36]. Whole blood incubated with antibodies and labelled with DiOC_6_ was perfused through a collagen-coated capillary at a shear rate of 1000 s^− 1^ and the resulting thrombi analysed by microscopy. Fig. 3 shows typical images of thrombi formed after 4 min perfusion in the presence of 10 μg/ml control antibody, or GOK/STIM1 or PM6/40. The GOK/STIM1 antibody showed a 26% inhibition of mean thrombus volume (*P* = 0.049, n = 5) (Fig. 4). The presence of the PM6/40 antibody reduced mean thrombus volume by 90% (*P* = 0.028; n = 3) confirming the total requirement of GPIB in mediating platelet adhesion to collagen under shear and validating the methodology of using antibody reagents in this system.

### 3.4 Effects of GOK/STIM1 antibody, the SOCE inhibitor BTP2 and non-SOCE inhibitor LOE908 on platelet aggregation

We then analysed effects on platelet aggregation mediated by TG (SOCE), 1-Oleoyl-2-acetyl-*sn*-glycerol (OAG; a non-SOCE activator), collagen and thrombin. We have shown that TG induces platelet aggregation by depletion of intracellular stores, and indomethacin partially reduces this response [3]. In the presence of indomethacin, the aggregation induced by TG is dependent on the presence of extracellular Ca^2 +^. We tested the effects of BTP2, a recently described inhibitor of SOCE that is specific at up to 1 μM but can reduce non-SOCE induced by OAG at 10 μM and above [16]. BTP2 inhibited TG-induced platelet aggregation in a dose-related manner with 1 μM reducing aggregation by 70% (*P* = 0.0007; Fig. 4A). At 1 μM, BTP2 did not significantly affect 60 μM OAG-induced aggregation but at 10 μM and above an inhibition was observed. We also tested the non-SOCE inhibitor LOE908, reported to inhibit Ca^2 +^ entry mediated by OAG which is an activator of TRPC6 and TRPC3 [14–17]. LOE908 at 10 (or 30) μM did not affect TG-induced platelet aggregation but almost completely reduced OAG-induced platelet aggregation (Fig. 4C, D). Thus BTP2 (at selective concentrations) and LOE908 could be used with caution to inhibit SOCE and non-SOCE respectively. The dialysed GOK/STIM1 antibody at 10 μg/ml (compared to a dialysed IgG2a [or PL/IM430, not shown]), did not inhibit aggregation induced by 5 μM TG (Fig. 4E, F) nor did it induce any response on its own (not shown).

These reagents were then tested against moderate concentrations of thrombin and collagen. The GOK/STIM1 antibody did not inhibit the shape change or the aggregation response by 0.5 U/ml thrombin (Fig. 4G), nor were these responses affected by the presence of 1 μM BTP2 or 10 μM LOE908 or both together (results not shown) (10 μM BTP2 did reduce thrombin-stimulated platelet aggregation but the effects could not be ascribed to inhibition of SOCE—results not shown). However against 10 μg/ml collagen, the GOK/STIM1 antibody caused a reduction in the aggregation by 23% (*P* = 0.0016; see Fig. 4G) with no effect if aggregation was induced by higher 20 μg/ml collagen concentration. BTP2 at 1 μM did not affect the aggregation recorded with either 10 or 15 μg/ml collagen; however LOE908 did reduce the extent of aggregation by 36% (*P* = 0.001; Fig. 4H), suggesting that the non-SOCE pathway contributed significantly to the aggregation response to collagen.

**Fig. 5:**
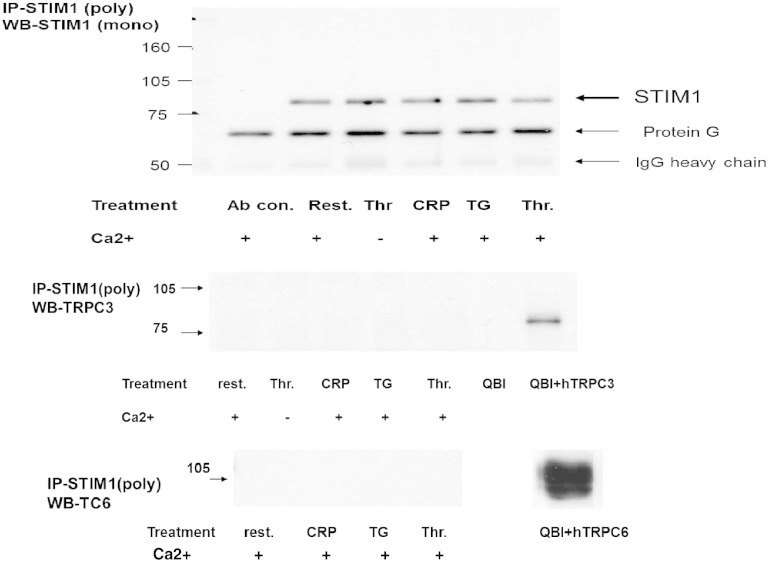
STIM1 does not co-immunoprecipitate TRPC6 or TRPC3 in human platelets. Platelets (1 × 10^9^ cells/ml) were treated with reagents as indicated for 1 min and lysates prepared. Immunoprecipitation was carried out with C terminal polyclonal antibody to STIM1, split into three and subjected to western blotting analysis in parallel. Immunoblots were probed with GOK/STIM1 (1/250 dilution), anti-TRPC6 (SL-TC6; 1/500 dilution) and anti-TRPC3 (A1978; 1/200 dilution) antibodies. QBI + hTRPC3 and QBI − hTRPC6 reflect lysates from hTRPC3 or hTRPC6 over-expressed in QBI-293 cells for positive identification of the respective channels. Blots are typical of three separate determinations.

Taken together, these results suggest that PM STIM1 may play a role in thrombus formation on a collagen-coated surface and collagen-induced platelet aggregation that may be independent of SOCE. To test if PM STIM1 interacted with components of the non-SOCE pathway we imunoprecipitated STIM1 to examine if TRPC6 or TRPC3 were co-immunoprecipitated. Fig. 5A shows that STIM1 can be avidly immunoprecipitated from resting and platelets treated with thrombin, CRP and TG using a polyclonal STIM1-C terminal recognising antibody from ProSci Inc. (USA). However when the immunprecipitates were immunoblotted with TRPC6 or TRPC3 antibodies there was no detection of these channels (Fig. 5B, C). TRPC6 and TRPC3 overexpressed in QBI-(HEK)-293 cells served as positive controls for detection. We were also unable to detect any co-immunopreciptation of TRPC4, TRPC5 or TRPC7 with STIM1 (results not shown). This suggests that PM STIM1 may modulate collagen induced platelet aggregation via a novel mechanism.

### 3.5 Identification of novel binding partners of STIM1 in human platelets

**Fig. 6:**
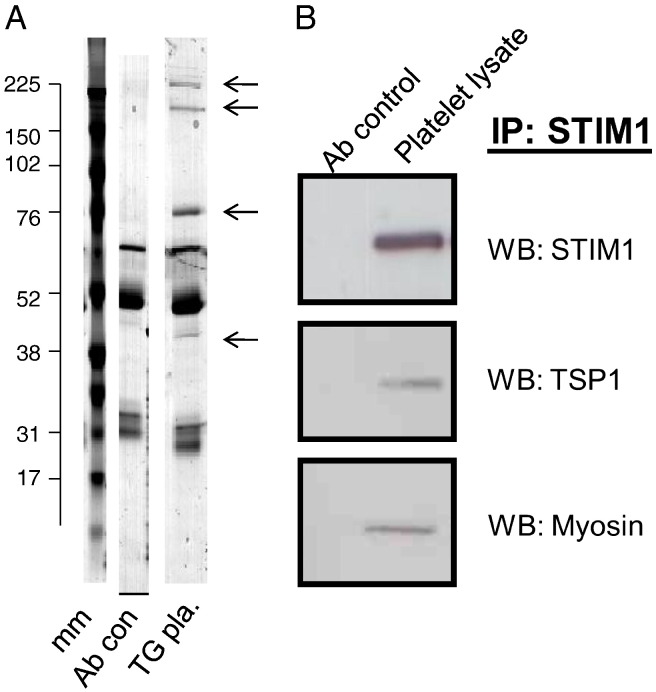
Immunoprecipitation of STIM1 for proteomic analysis and western blots. A. STIM1 was immunoprecipitated using an antibody recognising the C terminus of STIM1 from TG treated platelets (1 min) with the SDS-PAGE gels stained by Colloidal Coomassie stain (TG pla. Lane 3). Lane 1 mm = molecular markers; lane 2 Ab con = antibody reagent control including protein G; lane 3 STIM1 immunoprecipitate. Arrows reflect prominent bands immunoprecipitated by the STIM1 antibody. Bands and clear areas of the gel were used for peptide identification (see Materials and methods). Numbers on the left reflect the size of molecular markers in kDa. B. Co-immunoprecipitation of TSP1 and myosin with STIM1 from platelet lysates. WB-STIM1 probed with GOK/STIM1 (1/250 dilution), TSP-1 (1/200 dilution) and Myosin (1/1000 dilution) antibodies as indicated in Materials and methods. Ab control lane reflects a total reagent control with antibody, protein G etc. but without platelets.

To search for potential binding partners of STIM1 on the platelet surface we carried out a proteomic analysis of immunoprecipitated STIM1 from TG-treated platelets. The SDS-PAGE gel revealed protein bands additional to the antibody reagents that were visible with Coomassie staining (Fig. 6A). Proteins with 2 or more positive peptide hits returned from proteomic analysis are listed (Table 1). These include myosin-9, dedicator of cytokinesis protein-10 (DOCK10), thrombospondin-1 (TSP-1) and actin. All four proteins were confirmed to be present in platelet lysates by western blotting (results not shown). STIM1 peptides covered 38% of the sequence suggesting good immunoprecipitation. Coverage for myosin-9 was 3%, for DOCK10 1%, for TSP-1 11% and for actin 16%. As TSP-1 is a secreted protein of alpha granules and is known to bind to the plasma membrane; it may bind to PM STIM1. In contrast myosin-9, actin and DOCK10 are intracellular proteins and depending on their location may bind to ER or PM STIM1. We sought to confirm the co-immunoprecipitation using Western blotting. Fig. 6B shows that TSP-1 and myosin-9 could be detected as co-immunoprecipitated proteins with STIM1 from platelet lysates confirming them to be potential binding partners for STIM1.

## 4 Discussion

This study reports for the first time a potential supporting role of PM STIM1 in human platelet aggregation induced by collagen which is distinct from the SOCE role of STIM1 at the ER, and may involve interaction with the secreted protein TSP-1 in addition to cytosolic components such as DOCK10, myosin, actin and other components.

Although STIM1 in the endoplasmic reticulum is essential for sensing Ca^2 +^ depletion in the ER through the Ca^2 +^-binding N-terminal region [25,34] a fraction of STIM1 has been recognised to be expressed in the PM and suggested to participate in SOCE [35]. However, over-expression of N-terminally tagged STIM1 (to monitor STIM1 localisation) suggests that STIM1 does not insert into the PM [5,25]. Moreover, substitution of endogenous STIM1 with N-terminally tagged GFP-STIM1 in DT-40 cells fully supports SOCE, suggesting a non-essential role for PM STIM1 in Ca^2 +^ entry [5]. In platelets Lopez et al. reported the presence of STIM1 in the plasma membrane and a doubling of the surface expression with TG in a Ca^2 +^ independent manner [26]. However, the same group subsequently reported a 44% (at 10 s) and 28% (at 1 min) increase dependent on extracellular Ca^2 +^ [23]. In our studies an increase of STIM1 surface labelling upon store depletion was not observed and we favour migration of STIM1 into *punctae* as a mechanism to induce Ca^2 +^ entry rather than further insertion into the PM, as suggested by others [25]. Regarding a role in Ca^2 +^ entry the un-purified GOK/STIM1 antibody has been reported to inhibit Ca^2 +^ entry to various extents in various cells. Mignen et al. [30] reported that arachidonate regulated Ca^2 +^ (ARC) channels were more inhibited than CRAC channels. With platelets, Lopez et al. reported the neat un-purified GOK/STIM1 antibody (at 1 μg/ml vs no treatment) to inhibit Ca^2 +^ entry by 36% suggesting a supporting role [26]. However they recently suggest that PM STIM1 may mediate the inactivation of SOCE by extracellular Ca^2 +^ [23] and in an over-expression system suggesting that the EF hand domain and lysine rich domains are important in this function [22]. Our study reporting no effect on SOCE by the dialysed STIM1 antibody and the inhibition of SOCE by sodium azide, suggests caution with previous studies as the preservatives themselves may exert effects. Further concentrations of preservatives may vary between batches of the same or different antibody preparations. The sodium azide reduced cation entry mediated by TG, thrombin and convulxin, implicating azide to inhibit a common point in the SOCE pathway. In addition to other effects that may be relevant, sodium azide can inhibit complex IV of mitochondria [38] and inhibition of mitochondrial function has been shown to reduce Ca^2 +^ entry in many cells [19]. We did not examine the mechanism of azide action further as our purpose was to examine STIM1 function.

In our hands, surface binding of the STIM1 antibody reduced collagen-induced aggregation and mean thrombi volume formed on collagen-coated surfaces under flow. This suggests for the first time an extracellular role for STIM1 in a human platelet adhesion event which is independent of an effect on SOCE. On collagen-coated surfaces under flow, platelet attaches initially via the GP1B complex, becomes activated and thrombus formation ensues with further platelet recruitment to the adherent cells. Many adhesive receptors such as GPIB, GPVI, α2β1, and αIIbβ3 and signalling components have been shown to be important [21]. Antibodies to GP1B are particularly potent inhibitors of thrombus formation as shown with PM6/40. Engagement of PM STIM1 by the GOK/STIM1 antibody did not lead to a decrease of thrombus numbers but of thrombus volume, suggesting that the initial platelet attachment was not affected. The decrease of mean thrombus volume may therefore indicate a role for PM STIM1 in platelet–platelet interaction.

The effect of the GOK/STIM1 antibody on collagen-induced platelet responses supports the findings of others on STIM1-deficient mouse platelets which show reduced Ca^2 +^ entry to all agonists, but effects on aggregation responses to only collagen and CRP (not to ADP or thrombin) and reduced thrombus formation on collagen under flow [40]. Mouse platelets expressing an EF-hand mutation of STIM1 (Sax) exhibit increased basal Ca^2 +^ entry, and again reduced responses to agonists involving ITAM receptors but not G-protein-linked receptors suggesting a role of STIM1 in ITAM signalling [12]. Why ITAM signalling is preferentially effected by alterations in STIM1 is not known but may reflect a complex interaction of STIM1 with the GPVI signalling pathway that may include CLP36 which has been identified as a negative regulator of this pathway [13] and the proteins identified in this study. Interestingly, ADP- and thrombin-induced platelet aggregation is also normal in mouse platelets absent of Orai1 (clearly suggesting that SOCE is not required for this response [7,40]) in platelets absent of TRPC1[39] or TRPC6 [32] (suggesting that non-SOCE is also not required). Our finding that in human platelets BTP2 and LOE did not affect aggregation to thrombin supports the mouse studies. However this data is in contrast to work reported by Galan et al. where electroporation of the GOK/STIM1 or an anti-Orai1 antibody into human platelets inhibited ADP and thrombin induced platelet aggregation. Extracellular application of the GOK/STIM1 or an anti-TRPC1 antibody was also suggested to inhibit aggregation suggesting an interaction of PM STIM1, Orai1 and TRPC1 [11]. The reasons for the discrepancies are unclear [1,28]. Methodologically we have reported that electroporation of platelets allows free movement of molecules of less than 1000 Da across the platelet PM with no loss of cytosolic proteins [4,39] which is in contrast to the Galan study where a 150,000 Da antibody molecule is reported to gain access into the cytosol of platelets without loss of cytosolic proteins [11]. Additionally, we have been unable to confirm an interaction of STIM1 with TRPC1 in human or mouse platelets [39] nor with any other TRPC channel in human platelets by proteomics or Western blotting. Therefore we suggest the reduction in collagen induced aggregation by the STIM1 antibody and by the non-SOCE inhibitor LOE908 to be via distinct pathways.

As PM STIM1 has an EF-hand and a SAM domain facing the extracellular medium, PM STIM1 may take part in interactions involving other surface proteins. Our proteomic analysis of STIM1 pull-down extracts revealed novel binding partners of STIM1. Myosin-9 and actin are intracellular proteins involved with mobility and structural organisation of intracellular organelles and proteins. Binding to these cytoskeletal components may aid the membrane mobility of STIM1 during re-organisation into *punctae* after store depletion. The significance of STIM1 interaction with DOCK10 requires further study. The expression of DOCK10 in platelets was confirmed by western blotting (results not shown) and in a proteomic screen by Burkhart et al. [8]. DOCK10 is a known activator of cdc42 which is implicated in cell motility and lamellipodia formation. The observed co-immunoprecipitation of STIM1 with TSP-1 is of particular interest. TSP-1 has been associated with a number of stimulatory functions and reported to bind to surface proteins including CD36, CD47, β1, and β3 subunits of integrins and also to collagen itself. TSP-1 promotes aggregation [9,37] and antagonises NO and cAMP mediated effects in many cells including platelets though the pathways are yet to be fully established [20,33]. Interestingly, a patient was described whose platelets aggregated to several agonists except collagen and were found to lack GP1b and TSP-1 [24]. Re-addition of TSP-1 to the in vitro incubations restored the platelet response to collagen [24]. From our results we can hypothesise that PM STIM1 may bind TSP-1 and enhance GPVI signalling. Further a potential TSP-1, STIM1 and CLP36 interaction that may relieve the negative modulation of CLP36 on GPVI signalling is worthy of detailed study. In summary, this work suggests that PM STIM1 may play an important role in facilitating platelet activation by an interaction with surface-binding proteins that include TSP-1 in addition to intracellular components involved with collagen induced signalling. Further studies are required to elucidate the complex mechanisms involved.

## Disclosure of conflict of interest

The authors have no conflict of interest.

## Contributors

A Ambily, WJ Kaiser, C Pierro, EV Chamberlain, Z Lu, CI Jones and N Kassouf performed experiments. KS Authi and JM Gibbins designed the experiments and supervised the work. All authors were involved in writing the paper.

## Figures and Tables

**Table 1:** Analysis of STIM1 immunoprecipitations by proteomics. Only proteins with 2 or more peptide identifications are shown.

Protein ID and gene	Calculated MW (Da)	No. of peptides matched	% cov.	Peptide mass (Mr)	Peptide sequences identified
Myosin-9; MYH9_HUMAN Myosin-9, non-muscle. P35579	226,392	4	3%	1192.6088	ALELDSNLYR
				1868.9592	ANLQIDQINTDLNLER
				3016.2616	DLGEELEALKTELEDTLDSTAAQQELR
				1997.0541	KANLQIDQINTDLNLER
Dedicator of cytokinesis protein (DOCK)10; DOC10_HUMAN Dedicator of cytokinesis protein 10. Q96BY6	249,154	2	1%	1102.5982	AVSQLIADAGIGGSR
				1413.7576	LTGLSEISQR
Thrombospondin-1. TSP1_HUMAN Thrombospondin-1. P07996	129,300	10	11%	1206.6245	SITLFVQEDR
				1863.9037	MENAELDVPIQSVFTR
				1928.0228	IAKGGVNDNFQGVLQNVR
				1615.8067	GGVNDNFQGVLQNVR
				1393.7242	FVFGTTPEDILR
				2408.1386	DLQAICGISCDELSSMVLELR
				1245.6929	TIVTTLQDSIR
				1874.8911	FTGSQPFGQGVEHATANK
				1594.7376	QVTQSYWDTNPTR
				1008.524	AQGYSGLSVK
Stromal interaction molecule—1; STIM1_HUMAN Stromal interaction molecule 1. Q13586	77,375	19	38%	2031.844	ATGTSSGANSEESTAAEFCR
				2272.1158	IDKPLCHSEDEKLSFEAVR
				1511.8712	ALDTVLFGPPLLTR
				1324.6371	AEQSLHDLQER
				1437.8191	TVEVEKVHLEKK
				1070.6084	LRDEINLAK
				1682.9063	LRDEINLAKQEAQR
				1520.747	QKYAEEELEQVR
				1264.5935	YAEEELEQVR
				1873.8846	ELESHSSWYAPEALQK
				2326.0979	NTLFGTFHVAHSSSLDDVDHK
				1157.6404	QALSEVTAALR
				1605.8335	QRLTEPQHGLGSQR
				1321.6739	LTEPQHGLGSQR
				1815.7693	DLTHSDSESSLHMSDR
				1643.7321	AADEALNAMTSNGSHR
				2269.2682	LIEGVHPGSLVEKLPDSPALAK
				2345.107	AHSLMELSPSAPPGGSPHLDSSR
				1920.845	SHSPSSPDPDTPSPVGDSR
Actin, cytoplasmic; ACTG1_HUMAN Actin. P63261	41,766	4	16%	1953.178	VAPEEHPVLLTEAPLNPK
				1131.5197	GYSFTTTAER
				2230.0576	DLYANTVLSGGTTMYPGIADR
				1160.6111	EITALAPSTMK
